# Distress Responses in a Routine Vaccination Context: Relationships to Early Childhood Mental Health

**DOI:** 10.3390/children5020029

**Published:** 2018-02-21

**Authors:** Nicole M. Racine, Hannah G. Gennis, Rebecca Pillai Riddell, Saul Greenberg, Hartley Garfield

**Affiliations:** 1 Department of Psychology, University of Calgary & Alberta Children’s Hospital Research Institute, Calgary AB T2N 1N4, Canada; nicole.racine2@ucalgary.ca; 2 Department of Psychology, Faculty of Health, York University, The Opportunities to Understand Childhood Hurt Laboratory, 2006 Sherman Health Sciences Research Centre, Toronto ON M3J 1P3, Canada; hgennis@yorku.ca; 3 Department of Psychiatry Research, The Hospital for Sick Children, Toronto ON M5G 1X8, Canada;; 4 Department of Psychiatry, University of Toronto, Toronto ON M5S 1A1, Canada; 5 Department of Paediatrics, University of Toronto, Toronto ON M5S 1A1, Canada; saulped@yahoo.com (S.G.); hartley.garfield@gmail.com (H.G.); 6 Department of Paediatrics, The Hospital for Sick Children, Toronto, ON M5G 1X8, Canada

**Keywords:** preschooler, distress regulation, mental health, parent sensitivity

## Abstract

Social and emotional competencies, such as distress regulation, are established in early childhood and are critical for the development of children’s mental health and wellbeing. Routine vaccinations in primary care provide a unique opportunity to relate responses to a universal, relatively standardized, distress regulation paradigm (i.e., pain-related distress) to key developmental outcomes. The current study sought to examine distress regulation during routine vaccination in infancy and preschool as predictors of outcomes related to socioemotional competence in preschool. It was hypothesized that children with poorer distress regulation abilities post-vaccination would have lower socioemotional development. Furthermore, it was hypothesized that insensitive parenting would exacerbate this relationship for children with poor distress regulation abilities. As part of an ongoing longitudinal cohort, 172 parent–child dyads were videotaped during vaccinations in infancy and preschool, and subsequently participated in a full-day psychological assessment in a university lab. Videotapes were coded for child pre-needle distress (baseline distress), immediate post-needle pain-related distress reactivity (immediate distress reactivity), and pain-related distress regulation (distress regulation). Parent sensitivity during the preschool vaccination was also coded. Baseline distress prior to vaccination predicted greater externalizing problems and behavioral symptoms. Parent sensitivity did not moderate the association between any child distress behaviors and socioemotional development indicators. Child distress behaviors prior to injection, regardless of parent behavior, during the vaccination context may provide valuable information to health care professionals about child socioemotional functioning in the behavioral and emotional domains.

## 1. Introduction

Based on a large national study of nearly 250,000 Canadian children, 26% of preschool-aged children demonstrated vulnerability in at least one area of development, including the physical, social, emotional, cognitive, and communication domains at school entry [1]. Poor socioemotional development has been shown to put children at risk for low academic achievement as well as poor well-being throughout the lifespan [2]. Socioemotional competence in the first five years of life refers to a child’s ability to form close and secure relationships, experience and regulate emotions, and express emotions in appropriate ways [3]. Emotion regulation is one’s emerging ability to cope and recover both behaviorally and biologically from heightened levels of positive and negative emotions [4]. As part of emotion regulation, distress regulation can be understood in terms of one’s immediate behavioral response to a negative stimulus (distress reactivity) and one’s prolonged response over time (distress regulation). The foundations of distress regulation skills are laid in early childhood and poor development of these abilities put preschoolers at risk of mental health and academic difficulties both at school entry and later in life [5]. These long-term and profound implications stress the importance of identifying and providing remediation to children whose distress regulation skills are lagging. 

The development of distress regulation abilities occurs in the context of the parent–child relationship [2,6]. Although each child is born with unique biological and temperamental responses to positive and negative emotions, these responses do not necessarily confer risk on developmental outcomes when a child is in a supportive caregiving context [7]. However, when children are in less supportive or higher risk environments, poor emotion regulation can lead to poor developmental outcomes [8,9]. As such, caregiving is hypothesized to be a major mechanism that influences the association between distress regulation and children’s mental health and school readiness outcomes [7]. Regardless of temperamental predispositions, when children are appropriately scaffolded to learn how to manage their positive and negative emotions, they are more prepared to engage, attend, learn, and retain academic content [2]. In fact, recent research has shown that temperamentally sensitive children may benefit more from positive parenting environments than less temperamentally vulnerable children [10]. 

Due to the high levels of pain-related distress and the opportunity for parents to scaffold regulation of that distress, the vaccination context provides an excellent context to understand not only how children immediately react and regulate with respect to a very distressing stimulus, but also parent behaviors. Parent behavior in the vaccination context has been identified as one of the most robust predictors of child distress during preschool vaccination [11,12]. However, the relationship between caregiver sensitivity and child distress during vaccination with early socioemotional competence has not been explored. Given the highly predictive relationship with school readiness and future child development outcomes [13,14], finding behavioral predictors of socioemotional difficulties in a universal context that is generally standardized among children of a similar age (such as vaccination) would be a fruitful line of inquiry to better equip primary care practitioners to assess socioemotional competence in young children. Additionally, no studies to date have examined whether distress regulation during infancy is predictive of socioemotional development at the preschool age. Being able to identify children who may go on to have socioemotional difficulties would allow for early identification and intervention for these children. 

As a result of consistent and routine relationships with young children and their families, pediatric primary care providers are in a unique position to screen children who may be at risk for distress regulation problems, and thus subsequent socioemotional, behavioral, and academic difficulties [15]. In recent years there has been an increased impetus for mental health assessment and treatment to be provided in the primary care setting [16]. As such, the current paper seeks to identify ways in which children at risk of socioemotional difficulties can be identified within this framework of care. The vaccination context provides a repetitive situation where the child’s distress regulation abilities can be observed and monitored over time, as well as their caregiver’s response to the distress [17]. Given the aforementioned links between poor distress regulation and long-term mental health and academic difficulties, if distress regulation during routine preschool vaccination could be linked to measures of early socioemotional development including internalizing, externalizing, and behavior problems, this context may provide a new paradigm for supporting parent–child dyads that might be at particular risk for suboptimal child emotional and academic outcomes. Recent work has begun to demonstrate valid measures that screen for behavioral and emotional problems within primary care [18,19]. However, owing to the limitations of depending solely on parental reports [20,21], direct observation of self-regulatory abilities in this context would allow for a multi-modal and multi-informant screening perspective. The current study sets out to take the first exploratory step. 

The current study, using a large longitudinal vaccination cohort (the Opportunities to Understand Childhood Hurt Cohort/OUCH cohort [22]), sought to examine whether child distress regulation at 12 months and 4–5 years of age in the vaccination context (prior to a vaccination, immediate reactivity, and distress regulation) was predictive of early socioemotional development issues, including internalizing behavior (including anxiety, depression, and somatization), externalizing behavior (including hyperactivity, aggression, and conduct problems), and behavioral symptoms (including atypical behavior, withdrawal, and attention difficulties). We hypothesized that children with poorer distress regulation abilities would have greater internalizing, externalizing, and behavior problems. We also examined whether parent sensitivity during the vaccination procedure would predict early socioemotional development. We hypothesized that parent sensitivity would be positively associated with better socioemotional competence [23]. Finally, we examined whether parent sensitivity moderates the association between child distress regulation in the pain context and early socioemotional competence. We hypothesized that the association between poor distress regulation and poor socioemotional competence would be exacerbated for children of parents who demonstrated lower levels of sensitivity. 

## 2. Materials and Methods

### 2.1. Sample

The current investigation is part of a larger longitudinal vaccination cohort (OUCH Cohort) [22], which aims to examine the associations between infant and child pain-related distress and caregiver behavior, as well as broader children’s mental health outcomes. Participants were recruited between October 2007 and December 2015 from three pediatric clinics in Toronto, Canada. Infants and their caregivers were initially recruited at their 2, 4, and 6-month vaccinations and followed at multiple points over the first five years of life. The current analysis focuses on participants who were involved in both preschool waves (*N* = 172; in-clinic vaccination and full day lab assessment). No analyses within the current study overlap with previous work. A complete list of OUCH Cohort publications at [24].

Inclusion criteria for the original study were that caregivers could read and speak English, that the infants had no suspected developmental delays, impairments, or chronic illnesses, and had never been admitted to a neonatal intensive care unit. All children were considered developmentally typical, healthy, and low-risk. For the current analysis, parents were predominantly mothers (86%) with some fathers (14%), with an average age of 39.2 years (standard deviation (SD) = 4.12) at assessment. The children were 46.8% female (*n* = 80) and 53.2% male (*n* = 91) with an average of 4.83 years of age (SD = 0.59). 

The parents in our sample were highly educated, with 84.2% of mothers and 82.6% of fathers reporting a university-level education or higher. The sample was asked to report on their heritage culture during the infant vaccination wave of the OUCH cohort study. Our sample was culturally diverse, reporting a variety of heritage cultures (38.9% European, 16.8% North American, 12.6% Asian, 11.4% Jewish, 3.6% Latin/South American, 1.8% Middle Eastern/African, 11.4% mixed, and 3.6% other).

### 2.2. Procedure

Ethics approval was obtained from the Research Ethics Board (REB) at the affiliated university (Infant Wave REB Certificate Number: 2007-203; Approval Date: 16 October 2007; Preschool Wave REB Certificate Number: 2012-269; Approval Date: 22 November 2012). The methods for the vaccination procedure from the infant and preschool waves of the study have been reported elsewhere [11,22]. A brief overview of the methodology for the preschool vaccination and preschool psychological assessment follows. Parents who had previously participated in the OUCH study were given a flyer by a medical receptionist and asked whether they would like to learn more about a new study. If interested, informed consent was obtained and the parent completed a demographic information form. Ninety percent of approached parents agreed to participate. Two video cameras were used to capture both a close-up face shot and a wide shot of the parent and child for 5 min before the vaccination and 5 min after. This footage was coded for child pre-needle distress (baseline distress), immediate post-needle pain-related distress reactivity (immediate distress reactivity), pain-related distress regulation (distress regulation), and parent sensitivity. On average, each child received two vaccinations (Mean = 1.99, SD = 0.43) which were injected in each of the upper arms. At the preschool vaccination, children received their vaccinations uniformly positioned in a seated position. There were two primary pediatricians who performed the majority of injections, for which the procedure was standardized across children. 

Caregivers who participated during their child’s preschool vaccination were asked if they would be interested in participating with their child in a day-long assessment at the participating university (including a comprehensive battery of cognitive, emotional, behavioral, and academic achievement tests). Caregivers were informed that they would be provided with a psychological assessment report from a registered psychologist (R.P.R.) and a feedback session if requested or warranted. Caregivers who agreed to participate were scheduled for an assessment within eight weeks of the vaccination appointment. The psychological assessment took place at the OUCH laboratory at York University and was approximately 4–5 h in length. Each assessment was conducted by a doctoral trainee from a registered child clinical psychology program and was supervised by a registered psychologist and senior author (R.P.R). In compensation for their time, families were provided with a parking voucher, a $20.00 Canadian lunch voucher, and were provided with a psychological report within 3 months of the assessment. Fifty-seven percent of approached parents consented to participate in the assessment.

### 2.3. Measures

#### 2.3.1. Child and Parent Behavior during the Vaccination Appointment

##### Infant Baseline Distress, Distress Reactivity and Distress Regulation

The Modified Behavior Pain Scale (MBPS [25]) was used to assess infant pain-related distress 15 s prior to the vaccination, immediately after the vaccination, and 1 min after the vaccination (75 s after last needle) when the infant was 12 months of age. The 12-month time point was used as previous research has shown that it demonstrates the greatest variability in the first year of life [26]. The MBPS is comprised of three subsections including facial expression, cry, and body movement that were each coded by a behavior coder with a maximum score of 10. The MBPS has demonstrated moderate-to-high concurrent validity in the vaccination context [25]. In the current sample, interrater reliability was high, with intraclass correlations ranging from 0.93 to 0.96. 

##### Preschool Baseline Distress, Distress Reactivity, and Distress Regulation

The Face, Legs, Activity, Cry, and Consolability (FLACC) scale [27] was used to operationalize distress reactivity and distress regulation during the vaccination appointment at preschool. The FLACC is a behavioral rating scale that is a valid and reliable measure of procedural pain in infants and young children [27]. Higher scores indicate higher distress intensity. The FLACC is comprised of five behavioral indices: face, legs, arms, cry, and consolability, which are each rated using a scale from 0 to 2. The sum of these ratings is combined to obtain an overall score between 0 and 10 for each 15 s epoch. The FLACC scale was coded by trained coders, and interrater reliability coefficients for the current study were above 0.80. 

For the current study, baseline distress refers to a child’s response 30 s prior to their first needle (range 0 to 20). Immediate distress reactivity was operationalized as the child’s initial distress response to the needle for the first 0 to 30 s following the last needle in the vaccination procedure (range 0 to 20). Child distress regulation was operationalized as the child’s response for 30 s 1 min after the last needle had occurred ranging from 0 to 20 s (i.e., the epoch was 60–90 s after the last needle). 

##### Parent Sensitivity

The Maternal Behavior Q-Sort Short Version (MBQS [28]) was used to assess caregiver sensitivity during the preschool vaccination. The MBQS Short Version used in the current study is a 25-item version of the 90-item MBQS [29] used for measuring the quality of caregiving behavior during parent–child interactions. The MBQS full version was designed to be applicable to a wide developmental range (3 months to 5 years) and has recently been used with children up to 71 months of age [30]. The MBQS Short Version is reliable, has good construct validity, and is related to assessments using the full MBQS as well as to later cognitive development and attachment security [28]. In the current study, two coders who were trained by the scale developers independently coded the vaccination videos. Sixty-seven percent of the videos were double-coded (i.e., coded independently) and compared. For cases where coder scores differed by an absolute value of 0.2 or greater, the coders met, reviewed the videos and codes, and a code was reached by consensus. Inter-rater reliability was strong, with an intraclass correlation of 0.82.

#### 2.3.2. Child Self-Regulation Variables during the Psychological Assessment

##### Preschool Internalizing Behavior, Externalizing Behavior, and Behavioral Symptoms

Child internalizing behavior, externalizing behavior, and behavioral symptoms at the preschool psychological assessment were assessed using the Internalizing Problems, Externalizing Problems, and Behavior Symptoms Index composites from the Behavior Assessment System for Children-2, Parent Rating Scale-Preschool (BASC-2; Parent) [31]. The BASC-2 is a multi-dimensional assessment measure administered to parents in the form of a questionnaire and captures parent’s perception of the child’s socioemotional functioning. The child Internalizing Problems composite consists of the anxiety, depression, and somatization subscales, while the Externalizing Problems composite is comprised of the hyperactivity, aggression, and conduct problems subscales. The Behavior Symptoms subscale is comprised of the withdrawal, atypicality, and attention problems. Total raw scores for each scale are converted into composite scores. Scaled scores (T scores) were used in the current analysis with scores above 60 being considered in the at-risk range and scores above 70 considered in the clinical range. Psychometric properties for the BASC-2 are high with internal consistency and test re-test reliability above 0.80 [31]. 

### 2.4. Data Analysis

Exploratory correlation analyses and regression analyses were conducted using MPlus 8.0 (Muthén & Muthén, Los Angeles, CA, USA) and the Statistical Package for the Social Sciences (SPSS version 22, SPSS Inc., Chicago, IL, USA). The socioemotional variables had minimal missing data (<2%) and parent sensitivity had 18% missing data. Baseline distress, immediate distress reactivity, and distress regulation had less than 12% missing data for preschool and less than 16% for infancy. Analyses were performed with maximum likelihood, which allowed us to include 171 participants based on the missing at random assumption [32]. Maximum likelihood has been identified as an effective means of estimating missing data that reduces biases that often accompany list-wise deletion of participants with missing data [32]. One participant was not included because of missing data for all indicators included in the study. The maximum likelihood estimator with robust standard errors was used, which is robust to non-normality [33]. A statistical power analysis was performed for sample size estimation. Based on research that has demonstrated that moderators typically yield small effect sizes [34], an effect size of d = 0.15 was used to calculate sample size using G-Power (GPower 3.1 Software) [35]. With an *α* = 0.05 and power = 0.80, the projected sample size for a linear multiple regression with three predictors including the moderator was *N* = 119. Thus, our prosed sample size of 172 is adequate for the main objective of the study as well as the multiple comparisons that were conducted. 

Nine models were estimated in total, one for each of the child socioemotional development indicators (internalizing behaviors, externalizing behaviors, and behavior symptoms) at each of the three-time points (baseline distress, immediate pain reactivity, and pain regulation). Each linear regression model examined the predictive relationship of one of the child vaccination variables from preschool (baseline distress, immediate distress reactivity, distress regulation) as well as parent sensitivity on the given socioemotional development indicators. Given that there were no significant associations between the infant distress regulation variables and the child socioemotional development indicators, parsimonious models that simply included the preschool variables were estimated. To test whether parent sensitivity moderated the association between child vaccination behaviors and child socioemotional development, an interaction term was also entered in the model simultaneously. The pain regulation variable and maternal sensitivity variable were standardized. We planned to examine significant interactions with simple slope analyses. 

## 3. Results

### 3.1. Descriptive Statistics

Pearson correlations, means, and standard deviations (SD) for all variables are reported in Table 1. Infant baseline distress, immediate distress reactivity, and distress regulation were positively associated with each other. Child distress regulation indicators at the preschool time point were also positively associated with each other. Preschool baseline distress was positively associated with child externalizing behavior as well as behavior symptoms. Child internalizing behavior, externalizing behavior, and behavior symptoms were positively associated. 

### 3.2. Main Analyses

Results from the five regression analyses for each of the three pain-related distress outcomes (nine models in total) can be found in Table 2, Table 3 and Table 4. Beta weights provide a standardized indication of effect size. Two significant main effects were found: (1) externalizing behavior was positively predicted by baseline preschool pain-related distress at *p* ≤ 0.01 (*β* = 0.23); and (2) behavior symptoms were positively associated with baseline preschool pain-related distress (*β* = 0.17, *p* < 0.05). Parent sensitivity was not significantly associated with any of the child socioemotional indicators. Parent sensitivity was not a significant moderator in any of the models. 

## 4. Discussion

The current study sought to examine distress regulation during routine infant and preschool vaccination as a predictor of socioemotional development in early childhood. We hypothesized that children with poorer distress regulation abilities would have lower socioemotional functions with increased difficulties with internalizing behavior, externalizing behavior, and behavior symptom problems. Our hypotheses were partially supported whereby results revealed that higher distress prior to the vaccination at the preschool time point was associated with higher externalizing behaviors and behavior problems. We also examined whether caregiver sensitivity during the vaccination predicted early socioemotional development indicators. In contrast to our hypotheses, parent sensitivity was not associated with the child socioemotional development indicators nor did it moderate the association between children’s distress behaviors during the vaccination socioemotional competence. These results are discussed in the context of the existing literature and implications for clinical practice.

Our findings make a novel contribution in several ways. First, this is the first examination of child and parent behaviors during the well-child visit using a gold-standard assessment battery of socioemotional development measures. Previous work has examined these associations in the perioperative context and found that child behavioral distress was linked to externalizing behavior [36]. Our findings build on these studies by identifying child behavior during routine medical visits in the primary care setting, rather than rare occurrences such as operative procedures. As such, these findings provide greater clinical utility as difficulties with distress regulation can be identified by a provider who routinely interacts with the parent–child dyad and can make recommendations for referrals for intervention as needed. Overall, these findings are in line with a large body of work linking poor distress regulation in early childhood with behavioral and academic difficulties in preschool and the early school years [37]. 

Second, to date, no study has identified specific behaviors in the primary care context that can be observed by pediatricians and immunizing health professionals to identify children who may require additional support and skill development prior to school entry. Interestingly, we did not find a direct association between child distress regulation in the vaccination context and internalizing difficulties as measured in the current study. Among our socioemotional development indicators, externalizing behaviors (i.e., the lack of regulating disruptive behaviors) and behavior symptoms (i.e., failure to pay attention and withdraw from social situations) are more easily observed and identified than internalizing problems, which tend to be experienced internally. Given the short period of assessment afforded by the well-child visit, perhaps only socioemotional indicators with direct conceptual overlap are possible such as externalizing and behavior problems. Disruptive behaviors are a key school readiness outcome and it is very promising to be able to predict this from a child’s distress in anticipation of the painful vaccination [38]. Although clear variability exists [39], less variability in distress post-needle than pre-needle period may be the reason other relationships were not found. 

In the current study, parent sensitivity was not associated with socioemotional competence or distress regulation in the vaccination context. Caregiving is hypothesized to be the primary mechanism that impacts the association between child emotion regulation and later mental health outcomes [7]. Although we measured parent sensitivity in the current study, research has demonstrated that disrupted parenting (i.e., insensitive parenting), is one of the best predictors of poor developmental outcomes, including disorganized infant attachment [40] and behavioral outcomes in children [41]. In this low-risk sample, very few parents had clinically troubling levels of insensitivity, which may have contributed to our lack of findings. Future research should examine insensitivity in the immunization context to determine whether it is a better predictor of poor socioemotional development outcomes in children. 

Our findings should be interpreted in the context of some limitations. First, although a large longitudinal cohort was used in the current study, the majority of families who participated were of higher socioeconomic status with highly educated parents and high average household family incomes. As such, the generalizability of the current findings is reduced. Second, although the measures of cognitive and academic functioning in the current study were evaluated by an objective assessor, the socioemotional indicators were reported by parents. Third, on average there was a 3 month period between the child’s vaccination appointment and the psychological assessment. It is possible that other environmental factors or life events may have influenced the child’s internalizing or externalizing behaviors that were not accounted for in the current study, thereby decreasing the association among these variables. Finally, the sample in the current study was culturally diverse and thus cultural differences in both child behavior and parenting styles may have been present. Although not examined in the current study, future research should examine the influence of culture on the association between child distress regulation in the immunization context and future behavioral difficulties.

From a clinical perspective, the preschool vaccination context shows potential to better understand an important factor relating to children’s subsequent socioemotional functioning. The vaccination context provides a universal and generally standardized paradigm in which to observe distress behaviors that could be indicators of potential difficulties in transitioning to the school context. Being able to observe these behaviors in the primary care context, in conjunction with other primary care screening measures [18], could support offering parents information or avenues for support (e.g., psychoeducational groups, individual behavior therapy) to help moderate trajectories of children who may struggle with externalizing and behavior problems. 

## Figures and Tables

**Table 1:** Pearson correlations among variables.

	1	2	3	4	5	6	7	8	9	10
1. Baseline Distress 12 months	1									
2. Immediate Distress Reactivity 12 months	0.28 **	1								
3. Distress Regulation 1 Min 12 months	0.28 **	0.40 **	1							
4. Baseline Distress Preschool	0.15	0.22 **	0.11	1						
5. Immediate Distress Reactivity Preschool	0.03	0.19 *	0.04	0.54 **	1					
6. Distress Regulation 1 Min Preschool	−0.08	0.14	0.03	0.33 **	0.76 **	1				
7. Parent Sensitivity Preschool	0.07	0.03	−0.02	−0.17	−0.08	−0.07	1			
8. Internalizing Behavior	−0.11	0.01	0.09	0.09	0.15	0.10	0.07	1		
9. Externalizing Behavior	−0.09	−0.14	−0.04	0.21 **	0.13	0.01	−0.04	0.41 **	1	
10. Behavioral Symptoms	−0.11	−0.10	0.08	0.17 **	0.17 *	0.07	−0.03	0.66 **	0.80 **	1
Mean (SD)	3.27 (2.15)	8.11 (1.36)	5.61 (2.48)	5.46 (6.03)	8.22 (5.32)	4.77 (4.69)	0.36 (.40)	52.03 (9.78)	49.56 (7.37)	49.73 (7.62)

SD: standard deviation; * *p* < 0.05, ** *p* < 0.01.

**Table 2:** Baseline pain and parent sensitivity predicting socioemotional development at preschool.

	Internalizing Problems			Externalizing Problems			Behavior Symptoms		
	*β*	CI (95%)	*p*	*β*	CI (95%)	*p*	*β*	CI (95%)	*p*
Preschool Baseline Distress	0.11	−0.30, 0.24	0.12	0.23	0.07, 0.38	0.004	0.17	0.03, 0.32	0.02
Parent Sensitivity	0.08	−0.07, 0.24	0.30	−0.01	−0.17, 0.16	0.91	−0.001	−0.17, 0.17	0.99
Baseline Distress × Parent Sensitivity	0.04	−0.08, 0.16	0.54	0.16	−0.01, 0.32	0.06	0.06	−0.10, 0.22	0.43
R^2^	0.02			0.07			0.03		

CI: confidence interval; R^2^: R-squared; *β*: standardized regression coefficient.

**Table 3:** Immediate pain reactivity and parent sensitivity predicting socioemotional development at preschool.

	Internalizing Problems			Externalizing Problems			Behavior Symptoms		
	*β*	CI (95%)	*p*	*β*	CI (95%)	*p*	*β*	CI (95%)	*p*
Preschool Pain Reactivity	0.16	0.001, 0.31	0.05	0.12	−0.04, 0.29	0.14	0.16	−0.003, 0.33	0.05
Parent Sensitivity	0.08	−0.08, 0.24	0.32	-0.03	−0.20, 0.14	0.72	-0.01	-0.18, 0.16	0.88
Pain Reactivity × Parent Sensitivity	0.01	−0.15, 0.16	0.93	0.05	−0.12, 0.22	0.58	0.04	−0.14, 0.23	0.65
R^2^	0.03			0.02			0.03		

CI: confidence interval; R^2^: R-squared; *β*: standardized regression coefficient.

**Table 4:** Immediate pain regulation and parent sensitivity predicting socioemotional development at preschool.

	Internalizing Problems			Externalizing Problems			Behavior Symptoms		
	*β*	CI (95%)	*p*	*β*	CI (95%)	*p*	*β*	CI (95%)	*p*
Preschool Pain Regulation	0.10	−0.06, 0.25	0.21	0.01	−0.16, 0.18	0.93	0.07	−0.09, 0.23	0.41
Parent Sensitivity	0.07	−0.09, 0.22	0.40	-0.04	−0.21, 0.13	0.63	−0.02	−0.19, 0.15	0.79
Pain Regulation × Parent Sensitivity	−0.07	−0.23, 0.08	0.36	0.03	−0.14, 0.20	0.76	0.01	−0.16, 0.19	0.90
R^2^	0.02			0.003					0.01

CI: confidence interval; R^2^: R-squared; *β*: standardized regression coefficient.
